# Ability of SPI2 mutant of *S. typhi* to effectively induce antibody responses to the mucosal antigen enterotoxigenic *E. coli* heat labile toxin B subunit after oral delivery to humans

**DOI:** 10.1016/j.vaccine.2007.03.007

**Published:** 2007-05-22

**Authors:** S. Khan, S. Chatfield, R. Stratford, J. Bedwell, M. Bentley, S. Sulsh, R. Giemza, S. Smith, E. Bongard, C.A. Cosgrove, J. Johnson, G. Dougan, G.E. Griffin, J. Makin, D.J.M. Lewis

**Affiliations:** a Microscience, Wokingham Berkshire RG41 5TU, UK; b St. George's Vaccine Institute, London SW17 0RE, UK; c The Wellcome Trust Sanger Institute, Genome Campus, Cambridge CB10 1SA, UK

**Keywords:** Typhoid, Salmonella, Oral vaccine, ETEC, Enterotoxigenic *E. coli*

## Abstract

We have evaluated an oral vaccine based on an *Salmonella enteric* serovar typhi (*S*. typhi) Ty2 derivative TSB7 harboring deletion mutations in *ssaV* (SPI-2) and *aroC* together with a chromosomally integrated copy of *eltB* encoding the B subunit of enterotoxigenic *Escherichia coli* heat labile toxin (LT-B) in volunteers. Two oral doses of 10^8^ or 10^9^ CFU were administered to two groups of volunteers and both doses were well tolerated, with no vaccinemia, and only transient stool shedding. Immune responses to LT-B and *S. typhi* lipopolysaccharide were demonstrated in 67 and 97% of subjects, respectively, without evidence of anti-carrier immunity preventing boosting of LT-B responses in many cases. Further development of this salmonella-based (*spi*-VEC) system for oral delivery of heterologous antigens appears warranted.

## 1 Introduction

Enterotoxigenic *Escherichia coli* (ETEC), a significant cause of travelers’ diarrhea, is usually transmitted by the consumption of contaminated food and water arising from poor hygiene and sanitation. ETEC infection is endemic in the developing world and is also a major public health problem in children in these countries, causing an estimated 210 million cases of diarrhea and leading to approximately 380,000 deaths worldwide each year. Due to the debilitating effects of ETEC infections, considerable effort has been devoted to the development of a vaccine that will prevent ETEC diarrhea in humans.

Some of the strongest evidence supporting the supposition that an effective vaccine against ETEC can be developed comes from a large field study carried out in Bangladesh [1,2] using an oral vaccine against cholera. The vaccine consisted of whole cell killed cholera plus CT-B—a subunit of cholera toxin which cross-reacts with the equivalent subunit of the heat labile enterotoxin (LT-B) of ETEC. Two doses of the vaccine conferred protection against ETEC diarrhea in 73% of recipients for a period of 3 months. This was an important finding, not only providing evidence that immunization could confer protection against ETEC, but also demonstrating that the CT-B component alone provides protective immunity against ETEC disease through immunological cross-reactivity with LT-B.

It has already been demonstrated that several ETEC antigens including LT-B can be expressed in live attenuated *Salmonella* strains and immune responses can be elicited in animal models [3–7]. Here, we report the ability of a *S. typhi*-based oral vaccine, which may form the basis of a combined typhoid-ETEC travelers’ diarrhea vaccine, to deliver an ETEC antigen to the humoral immune system in humans.

## 2 Material and methods

### 2.1 Study design

The study was open label, with sequential, dose-escalating cohorts, and not placebo controlled. The study was conducted under a Clinical Trial Exemption from the UK Medicines Control Agency, now known as The Medicine Healthcare Products Regulatory Agency and permission for Deliberate Release of a GMO was obtained from Department for the Environment Food and Rural Affairs (DEFRA). Primary ethical permission was obtained from the Wandsworth Local Research Ethics Committee, and permission to enroll students from Kingston University was granted by the Kingston University Local Research Ethics Committee. Written informed consent was obtained after submission of a detailed explanation of the protocol. Subjects were excluded if they had a sensitivity to ciprofloxacin, trimethoprim-sulphamethoxazole or related antibiotics, had a known immunodeficiency or were on immunosuppressive therapy, had a history of gallbladder or gastrointestinal disease or some other significant medical condition, had received immunization against *Salmonella* in the preceding 5 years, or had an anti-*S. typhi* O-antibody titer in serum as determined by enzyme-linked immunosorbent assay (ELISA) that was >3 standard deviations above the mean of a group of healthy controls. Subjects were also excluded if they had household contacts who were at risk for transmission of vaccine bacteria.

Vaccines were manufactured according to Good Manufacturing Practice protocols by Eurogentec Ltd. (Liege, Belgium) and were derived from a tested and characterized Master Cell Bank, also manufactured according to cGMP protocols. The vaccine was packed by dose (10^8^ or 10^9^ CFU). Each dose of vaccine was frozen in a 1.5 ml cryo-vial. The vials of the vaccine were stored at −80 ± 5 °C, until required for administration. Prior to administration, each dose of vaccine was be made up to 50 ml with 2% (w/v) sodium bicarbonate solution no more than 30 min before dosing orally to volunteers.

Thirty-six volunteers were recruited (age range, 18–50 years). After fasting for at least 2 h, the volunteers swallowed 100 ml of 2% (w/v) sodium bicarbonate solution to buffer gastric acid and then received a single dose of either 10^8^ or 10^9^ CFU of ETEC Vaccine 1, on two occasions, 56 days apart (i.e. vaccine administered at the same dose level on two occasions 56 days apart). Volunteers remained at the investigative site, under observation, for 48 h following each immunization (pulse, blood pressure and respiratory rate were measured prior to immunization and at 30, 60 and 120 min after immunization) and then followed up daily for 1 week and weekly for 1 month post-immunization. Volunteers were assessed for reactogenicity and other adverse events by physical examination and by the completion of diary cards.

Subjects received the vaccine in three sequential groups of 12 subjects (Groups 1, 2 and 3). Due to constraints on the number of subjects that could be accommodated at any one time at the site, each group was vaccinated in two cohorts of six subjects. Group 1 (comprising cohorts 1 and 2) received a dose of 10^8^ CFU. The dose level in Group 2 (comprising cohorts 3 and 4) depended on the safety profile observed at the first (10^8^ CFU) dose. The dose level in Group 3 (comprising of cohorts 5 and 6) depended on the safety profile of the previous two groups. The intention was to dose escalate to 10^9^ CFU in Group 2 and 3. Subjects were administered the second dose of the vaccine after a satisfactory evaluation of safety at 14 days post administration of the first dose.

### 2.2 Construction of *S. typhi* (Ty2 *aroC*^−^*ssaV*^−^*ssaG*^−^*eltB*) TSB7 (ETEC Vaccine 1)

The construction of the vaccine strain in ETEC Vaccine 1, *S. typhi* (Ty2 *aroC*^−^ *ssaV*^−^ *ssaG*^−^ *eltB*) TSB7 has been described previously [7]. Briefly, the *ssaG*^−^ *eltB* fusion was introduced into the chromosome of *S. typhi* ZH9 (Ty2 Δ*aroC* Δ*ssaV*) at the site of the attenuating *aroC* deletion by transformation with the suicide vector construct pCVD442/*ssaG*^−^ *eltB*. One strain, termed *S. typhi* TSB7 (Ty2 Δ*aroC* Δ*ssaV ssaG*^−^ *eltB*), was selected for further study. The insertion of the *ssaG*^−^ *eltB* into the *aroC* deletion of *S. typhi* ZH9 (Ty2 Δ*aroC* Δ*ssaV*) and the integrity of the *ssaV* deletion were confirmed by PCR, Southern blot and sequence analysis.

### 2.3 Microbiological, hematological, biochemical, and clinical monitoring

Blood, stool, and urine cultures were collected before immunization on day of immunization, daily for 7 days, and weekly for 28 days after the administration of the first and second dose of the vaccine. According to standard United Kingdom Public Health practice, persons infected with non-typhoidal *Salmonella* should be considered non-infectious when two separate stool samples are negative on culture. The protocol stated that, if a volunteer demonstrated (beyond Day 7) persistent fecal shedding of *S. typhi*, then oral ciprofloxacin would be administered, and the volunteer monitored until two consecutive samples were negative.

Blood, stool and urine cultures were performed by the routine pathology service of St. George's Hospital. Fecal samples were inoculated onto desoxycholate citrate agar plus aromatic supplements (DCAaro) and a small aliquot emulsified in selenite F plus aromatic supplements (SFaro) enrichment broth. After overnight incubation at 37 °C, one loopful of SFaro broth was subcultured onto a fresh DCAaro plate to obtain discrete colonies. Equal volumes of urine and double strength SFaro broth were incubated at 37 °C for 24 h. A loopful of broth was removed and inoculated onto DCAaro plates. Five milliliter of blood was inoculated into aerobic and anaerobic bottles, maintaining a blood/broth ratio of 1:10 and incubated at 37 °C by using the Organon Teknika BacT/Alert blood culture system. Blood cultures were continuously monitored for 5 days, and a gram stain was performed on all bottles triggering a positive reaction. Those containing gram-negative bacilli were subcultured onto xylose lysine desoxycholate agar (XLD). Non-lactose-fermenting isolates on XLD and negative by oxidase test were characterized for biochemical reaction by using the Analytical Profile Index system (bioMérieux). Organisms identified as *Salmonella* were serotyped by using *Salmonella* agglutinating antisera (PROLAB and Central Public Health Laboratory, Colindale, UK). Urea and electrolytes, liver function tests, full blood count, erythrocyte sedimentation rate were measured in the routine pathology laboratories of St. George's Hospital. Volunteers kept a daily record of symptoms for 28 days post each dose and 7 days prior to each dose, including frequency and consistency of stool, headache, fever, nausea, vomiting, and other constitutional upset.

### 2.4 ELISA and ELISPOT assays for *S. typhi*-LPS specific serum IgG antibody and IgA ASC responses

Blood samples were collected prior to immunization and then on Days 7, 14, 21, 28, after each dose for the enzyme-linked immunosorbant assay (ELISA). For the enzyme-linked immunospot (ELISPOT) assays blood samples were collected prior to immunization and then on Days 7, 11, 14 (not for LPS), after each dose. Serum was frozen at −70 °C until analysis by ELISA. Peripheral blood mononuclear cells (PBMCs) were collected from the volunteers and assayed for the presence of specific antibody-secreting cells (ASCs) by using the ELISPOT technique. Briefly, PBMCs obtained by venepuncture were isolated by Ficoll–Paque centrifugation. PBMCs in concentrations of 2.5 × 10^5^, 5.0 × 10^5^ and 1 × 10^6^ cells in 100 μl of RPMI 1640/Glutamax/FBS/Penicillin/Streptomycin for each sample to be tested were added to wells of 96 well Multiscreen-IP nitrocellulose microtiter plates, which were either uncoated (control wells) or previously coated for 120–130 min at 37 °C with 5 μg/ml lipopolysaccharide, *S. typhosa* (*S. typhi*) (Sigma Chemical L-6386) in Reggiardo's Buffer plus 0.1% sodium deoxycholate and unbound binding sites blocked with PBS/3% BSA. Cells were incubated overnight at 37 °C in 5% CO_2_. After incubation the plates were washed and wells received 100 μl of phosphatase-labelled goat anti-human IgA antibody diluted 1:1000 in phosphate buffered saline (PBS). After 60–70 min incubation at 37 °C with gentle shaking, wells were washed, and spots were then visualized by the addition of NBT (nitroblue tetrazolium)-BCIP (5-brom-4-chloro-3-indolylphosphate) and enumerated under low power magnification. To account for non-specific binding of the cells to the wells of the plate, the mean number of spots in uncoated wells was subtracted from the mean number of spots in antigen-coated wells to give a mean number of ASCs. The mean of the number of ASCs (adjusted for dilution factor) for the three dilutions of each sample was determined and expressed as ASCs/10^6^ PBMCs.

For the ELISA, the same reagents and dilutions for coating Immunlon 4HBX plates were used as in the ELISPOT assay. Replicate 100 μl aliquots of serum diluted in PBS/1% BSA were added to the antigen coated and uncoated wells, serially diluted across the plate. Each plate contained dilutions of positive control sera from volunteers vaccinated with Vivotif (Berna). After 1 h incubation at 37 °C plates were washed and 100 μl of rabbit anti-human IgG (DAKO A0423) was added to each well. Plates were incubated at 37 °C for 60 min, washed and then 100 μl of goat anti-rabbit IgG alkaline phosphatase conjugate (ISL 075-1506) added to each well and the plate incubated for a further 60 min at 37 °C. Anti-LPS IgG activity was detected by incubating the wells for 30 min at room temperature with pNPP substrate and the absorbance read at 405 nm. The antibody titer was defined as the dilution required to reduce the signal for a given sample to background levels, and for each timepoint the titer was compared against day zero serum run simultaneously to calculate a fold increase.

### 2.5 ELISA and ELISPOT assays for heat labile toxin (LT) specific serum IgG antibody and IgA ASC responses

PBMCs were prepared from fresh blood and diluted in the same concentrations as for the ELISPOT assay for *S. typhi*-LPS ASC response described previously and added to wells of 96 well Multiscreen-IP nitrocellulose microtiter plates, which were either uncoated (control wells) or previously coated for 2 h at 37 °C with 5 μg/ml of purified heat labile toxin (LT) from *E. coli* (Berna Biotech Custom Batch) in carbonate coating buffer. Cells were incubated overnight at 37 °C in 5% CO_2_. After the plates were washed, wells received 100 μl of goat anti-human IgA-alkaline phosphatase labelled antibody (Immune systems Ltd. 075-1001) diluted 1:1000 in 1% BSA/PBS. After 1 h incubation at 37 °C, wells were washed and spots were then visualized by the addition of NBT-BCIP and enumerated under low power magnification. The number of ASCs was calculated as described previously and expressed as ASCs/10^6^ PBMCs.

Monosialoganglioside GM_1_ (GM_1_) has a high binding affinity for heat labile toxin (LT) and was exploited in the assay to detect anti-heat labile specific IgG. Hundred microliters of GM_1_ (0.5 μg/ml) in Reggiardo's buffer plus 0.1% sodium deoxycholate was added to wells of Immulon 4HBX (96 wells) microtiter plates and incubated overnight at 37 °C. The unbound binding sites were blocked with PBS/3% BSA and after washing, 100 μl of LT (1.0 μg/ml in PBS) was added to the designated coated wells. Plates were incubated for 1 h at 37 °C. After washing, replicate 100 μl serum samples diluted in PBST/1% BSA were added to the coated and uncoated wells, serially diluted across the plate. Each plate contained dilutions of positive control sera from volunteers vaccinated with Dukoral (SBL). After 1 h incubation at 37 °C plates were washed and 100 μl of goat anti-human IgG (Southern Biotech 2040-01) was added to each well and incubated for 1 h at 37 °C. After washing 100 μl of rabbit anti-goat IgG HRP conjugate (ISL 14-13-06) was added to each well and the plate incubated for a further 60 min at 37 °C. Anti-LT IgG activity was detected by incubating the wells for 10 min at room temperature with OPD substrate and the absorbance read at 405 nm. The antibody titer was defined as the dilution required to reduce the signal for a given sample to background levels, and for each timepoint the titer was compared against day zero serum run simultaneously to calculate a fold increase.

### 2.6 Definition of a positive response in ELISA and ELISPOT

For serum IgG antibody responses, Day 0 serum was run in the ELISA for each antigen at each time point after immunization and 4-fold or greater increase from Day 0 in antigen-specific serum IgG on either Day 7,14, 21 28, or 56 was taken as a response to the first immunization; and on Days 63, 70, 77, 84 as a response to the second. For the ELISPOT an increase above baseline in the number (to 10 or more) of ASCs per 10^6^ PBMCs which secreted antigen-specific IgA on day 7 or 11 or 14 after each vaccination was considered a positive response.

### 2.7 Statistical analysis

Analyses were performed as the number and percentage of subjects with an immune response concluding with a two-sided 95% exact CI. The *p*-value from two-sided Fisher's Exact Test at the 5% significance level (testing against an assumed immune response of 50%) was also calculated.

## 3 Results

### 3.1 Clinical monitoring

The ETEC Vaccine 1 was extremely well tolerated. There were no serious vaccine-related adverse events. All vaccine-related adverse events were transient and mild-moderate severity. There was no dose–response in the frequency of adverse events. As with other oral immunization studies employing bicarbonate buffer to neutralize gastric acid, mild to moderate and transient gastrointestinal symptoms were commonly recorded. Only one subject recorded gastrointestinal cramps graded as ‘severe’ which occurred 7 days after ingesting the first dose of 10^8^ CFU. Table 1 lists the frequency of gastrointestinal adverse events post-immunization. Two subjects withdrew from the study, both in the 10^9^ CFU group and both before the second dose was given. One subject withdrew consent, and another was withdrawn from the study due to epigastric pain 53 and 56 days after the first immunization, thought to be caused by a suspected peptic ulcer.

Ten subjects reported a fever at some time during the study period, but there was no obvious pattern for the timing of fever in relation to the dosing, except one subject who reported a fever on Day 3 after first dose of 10^9^ CFU and this was the only event of fever that could possibly be related to the administration of the vaccine. All events of fever were mild in nature, except one event of a moderate fever on Day 10 after the first dose of 10^8^ CFU.

There were no vaccine-related and clinically-significant abnormalities of hematological or biochemical parameters during the study.

### 3.2 Microbiological monitoring

No subjects developed bacteraemia post-immunization. The fecal shedding profile was consistent with that described previously [8], with the majority of subjects exhibiting only transient shedding within the first 3 days following immunization (Table 2). Only one subject shed the vaccine beyond Day 6, with isolated fecal shedding on Day 14. The frequency and pattern of fecal shedding is shown in Table 2. There were no obvious differences in the duration or time of onset of stool shedding, between the high and low dose groups, or for first and second immunizations. These data suggest that the shedding observed represents transient passage of bacteria and not sustained colonization.

### 3.3 Immune responses

Table 3 shows the immune responses detected by ELISPOT or ELISA separately for subjects completing the protocol, at the different dose levels and for each individual antigen.

*For the responses to S. typhi lipopolysaccharide*: The vaccine induced frequent and high level responses to *S. typhi* lipopolysaccharide, as described previously in trials [9] of the parent strain *S. typhi* ZH9 which lacks the *ssaG*^−^ *eltB* fusion. Taking all 36 subjects recruited on as an intention-to-treat population, 97% demonstrated an IgG or IgA immune response against *S. typhi* lipopolysaccharide measured either by ELISPOT or ELISA, and the majority of responders had both an IgG response (89%), as measured by ELISA, and an IgA response (92%), as measured by ELISPOT. When the observed response rates to *S. typhi* lipopolysaccharide were tested against an assumed response rate of 50%, for all tests, the *p*-values for the proportions of responders were <0.05, showing that the responses observed could be confidently declared to be different to the assumed response of 50%. Thus, the insertion of the *ssaG*^−^ *eltB* fusion did not effect the ability of ETEC Vaccine 1 to induce high level responses against *S. typhi* lipopolysaccharide.

*For the responses to ETEC LT*: Amongst the 36 subjects recruited, 67% demonstrated an IgG or IgA immune response against ETEC LT measured either by ELISPOT or ELISA. No dose–response was apparent with the same percentage of subjects in each dose group developing an immune response (10^8^ CFU: 8/12, 67%; 10^9^ CFU: 16/24, 67%). The majority of the responders in each group had an IgG response (22 subjects overall, 61%), as measured by ELISA, but few subjects (4 subjects overall, 11%) had an IgA response, as measured by ELISPOT. For both vaccine groups, and for all subjects combined, the observed response rates to LT were tested against an assumed response rate of 50%. However, for all tests, the *p*-values for the proportions of responders were not statistically significant, showing that the responses observed could not be confidently declared to be different to the assumed response of 50%. However, in the UK population it would be extremely unlikely for a subject to seroconvert to ETEC LT, as this organism is not present in the environment, and so a 50% seroconversion rate is probably too strict.

*For subjects receiving 10*^*8*^* CFU*: After first immunization mean peak IgG titer to LT 2625 (peak fold increase 2.5), mean peak IgA ASC response to LT 0, mean peak IgG titer to LPS 2073 (15.4), mean peak IgA ASC response to LPS 121. After second immunization mean peak IgG titer to LT 4743 (peak fold increase 3.7), mean peak IgA ASC response to LT 0.9, mean peak IgG titer to LPS 3672 (16.5) mean peak IgA ASC response to LPS 15.7.

*For subjects receiving 10*^*9*^* CFU*: After first immunization mean peak IgG titer to LT 4184 (peak fold increase 4.2), mean peak IgA ASC response to LT 8.29, mean peak IgG titer to LPS 3724 (13.1), mean peak IgA ASC response to LPS 158. After second immunization mean peak IgG titer to LT 3056 (peak fold increase 3.5), mean peak IgA ASC response to LT 0.73, mean peak IgG titer to LPS 1680 (7.9), mean peak IgA ASC response to LPS 13.

Given the small number of subjects studied at each dose level, and the wide scatter in the responses, interpretation of the correlation between responses measured in the two assays to the two antigens must be interpreted with caution. Non-parametric Spearman correlation with two-tailed *p*-values, was employed using GraphPad Prism as a preliminary investigation of the effect of the carrier response on the response to the heterologous antigen.

A positive correlation was observed between the peak serum IgG response to *S. typhi* LPS after the first immunization and the peak serum IgG response to *S. typhi* LPS after the second immunization, and the peak ASC IgA responses to *S. typhi* LPS after the first but not the second immunization (Spearman correlation coefficients: 0.89, 0.62, −0.06; *p*-values: <0.0001, <0.0001, 0.75, respectively). There was also a positive correlation between the peak serum IgG response to *S. typhi* LPS after the first immunization and the peak ASC IgA responses to ETEC LT after the first but not the second immunization (Spearman correlation coefficients: 0.43, 0.27; *p*-values: 0.009, 0.12, respectively). There was no correlation in the peak serum IgG response to *S. typhi* LPS after the second immunization and the peak ASC response to LPS after the second immunization (Spearman correlation coefficient: 0.06; *p* = 0.71). As there was a correlation between the serum IgG responses to first and second immunizations, these observations raise the possibility that the ASC responses to LPS may have followed a different time course after the second immunization, resulting in the peak response being missed, rather than immunity from the first immunization blocking a response to the second.

A positive correlation was observed between the peak serum IgG response to ETEC LT after the first immunization and the peak serum IgG response to ETEC LT after the second immunization, and with the peak ASC IgA responses to ETEC LT after the first and second immunizations (Spearman correlation coefficients: 0.89, 0.60, 0.59; *p* values: <0.0001, <0.0001, 0.0002, respectively). There was also a positive correlation between the peak serum IgG response to ETEC LT after the second immunization and the peak ASC IgA responses to ETEC LT after the first and second immunizations (Spearman correlation coefficients: 0.52, 0.55; *p* values: 0.002, 0.0006, respectively). There was no correlation in the peak serum IgG response to ETEC LT after the first or second immunizations and any of the responses to *S. typhi* LPS. These data suggest that the response to LPS did not affect the response to ETEC LT, and that the first immunization tended to prime for the second immunization to ETEC LT both in regard to serum and IgA ASCs. There also appears to have been less effect of the first immunization on the timing ASC response to the second, possibly indicating a less powerful priming for LT compared with LPS.

## 4 Discussion

With an estimated 210 million cases of ETEC-induced diarrhea and 380,000 deaths worldwide each year [10], a vaccine that will prevent or ameliorate the severity of ETEC infection in humans is urgently required. The major pathogenic mechanisms of ETEC include colonization of the small intestine and elaboration of one or more enterotoxins that through various mechanisms may induce water and electrolyte secretion resulting in diarrhea [11,12]. As part of the colonization process, ETEC possess specialized pili that act as ligands to bind the bacterial cells to specific complex carbohydrate receptors on the epithelial cell surfaces of the small intestine. Since this interaction results in colonization of the intestine by ETEC, with subsequent multiplication on the gut surface, these pili are termed colonization-factor antigens (CFA). Numerous CFA have been identified and there is considerable diversity in the expression of these across different clinical isolates [13–15]. These CFA enable ETEC to overcome the peristaltic defense mechanism of the small intestine in order to colonize the bowel. Following colonization, ETEC–enterotoxins stimulate excessive electrolyte and fluid secretion, primarily from the small intestine crypt cells, by inducing increased formation of cyclic adenosine monophosphate (AMP) and/cyclic guanosine monophosphate (GMP) in the epithelial cells [10,16].

ETEC may produce either or both of a heat labile enterotoxin (LT) and a heat stable enterotoxin (ST). LT is structurally, functionally and immunologically closely related, although not identical, to cholera toxin (CT). LT consists of five copies of the B subunit and one copy of the A subunit and both these proteins cross-react immunologically with the corresponding CT subunit proteins. The anti-LT immune response is mainly directed against the B-subunit portion of the molecule [10,16]. The demonstration that an oral vaccine consisting of whole cell killed cholera plus CT-B [1,2] can provide protective immunity against ETEC provides a strong rationale for the development a subunit vaccine against ETEC based on LT. The oral whole cell/CT-B vaccine was further evaluated in Finnish travelers going to Morocco, and found to confer 60–70% protection [17]. Based on these data the whole cell cholera vaccine has been licensed in Sweden and Norway as an ETEC vaccine (Dukoral^®^).

As wild-type ETEC strains express a large variety of colonization factors, a vaccine based on the conserved LT-B molecule is attractive. Therefore, the findings reported above have stimulated considerable interest in the potential for developing LT-B as a travelers’ vaccine. Antibodies to LT are also protective against ETEC in animals [18]. There are several vaccines against travelers’ diarrhea caused by ETEC in development. One of the most promising approaches is the use of live attenuated vaccines given by the oral route, either based on attenuation of the ETEC strains directly, or using a live attenuated vector such as *Salmonella* to deliver ETEC antigens. Live attenuated organisms can be developed to be highly effective vaccines; immune responses elicited can often be of greater magnitude and of longer duration than those produced by non-living antigens [19,20].

Because of their intimate interaction with the gut associated lymphoid tissue (GALT), live attenuated *Salmonella* make attractive vectors for the delivery of heterologous antigens from other pathogens. They can also be given orally and live *Salmonellae* are known to stimulate potent humoral, cellular and secretory anti-*Salmonella* responses against co-expressed heterologous antigens [21], including LT-B in animal models [3–7]. We have previously tested a live attenuated *S. typhi*-based oral typhoid vaccine in humans, named *S. typhi* (Ty2 *aroC*^−^ *ssaV*^−^) ZH9 [8]. This strain was subsequently used as a vector to express LT-B, which resulted in potent anti-LT responses in mice following intranasal and subcutaneous administration [7,22]. This *S. typhi* vector harbors independently attenuating deletion mutations in the *aroC* and *ssaV* genes. The *aroC* gene encodes chorismate synthase, an enzyme involved in the biosynthesis of aromatic compounds [23,24]. The *ssaV* gene encodes a component of the type III secretion system encoded on *Salmonella* pathogenicity island 2 (SPI-2) [25]. In addition, a SPI-2-associated *in vivo* inducible *ssaG* promoter was used to drive the expression of LT-B [24]. Also, in order to enhance the stability of the construct in vivo the promoter/antigen fusion was inserted into the *S. typhi* chromosome [7,26].

In this study, we found that ETEC Vaccine 1 was able to reliably induce both serum IgG and mucosal IgA responses to *S. typhi* lipopolysaccharide in over 95% of subjects at both the low and high dose levels—a crucial characteristic of a protective vaccine against typhoid, and in keeping with our previous experience of the parent strain. This also indicates that the insertion of the LT gene has not compromised the fitness of the organism in its ability to establish a vaccine take and induce both systemic and mucosal immunity, a major attraction of orally delivered live attenuated vaccines.

Generally, live attenuated *Salmonella* have been somewhat disappointing in their ability to induce immune responses to heterologous antigens after oral delivery to humans, in contrast to their immunogenicity in murine models. In addition there may be a discordance between mucosal IgA responses detected by ELISPOT and serum IgG antibody. For example, while oral administration of CVD 909 (a live attenuated *S. typhi* strain engineered to constitutively express the *S. typhi* capsular antigen Vi), induced detectable levels of Vi specific IgA antibody-secreting cells, in the majority of human volunteers only 2/32 recipients generated serum anti-Vi IgG antibody [27]. Furthermore, while a prototype live attenuated *S. typhi* vaccine engineered to express the urease antigen of *Helicobacter pylori* [28,29], induced T cell priming in around 56% of volunteers receiving one or more dose orally, no serological responses to the heterologous antigen could be detected. Therefore, our observation that 67% of all volunteers demonstrated an IgG or IgA immune response against ETEC LT measured either by ELISPOT or ELISA, independent of dose level, is the first demonstration in a human trial to our knowledge that a recombinant *S. typhi* can effectively prime for a humoral response to a heterologous antigen in humans. The fact that only a single, integrated copy of the LT gene is present in the strain, rather than multicopy plasmid expression, makes the immune responses observed even more significant and demonstrates the utility of the *ssaG* promoter in driving immunologically relevant levels of antigen. The lack of a carrier effect, whereby immunity to the carrier inhibits immune responses, as demonstrated in this study by responses to both first and second immunizations is also encouraging.

Our observation that ETEC Vaccine 1 was well tolerated, relatively unreactogenic, did not cause vaccinemia, and induced only transient low level shedding in volunteers is crucial for further clinical development. In particular, the low level of stool shedding is an important consideration, as release into the environment is a key feature of genetically modified organism identified by regulatory authorities. Furthermore, the lack of enhanced shedding or reactogenicity by the insertion of the LT-B protein is significant, as theoretically the presence of LT-B on the surface of the organism could enhance gut binding to GM1 gangliosides area. The lack of appreciable side effects combined with the induction of anti-LT specific immune responses in the majority of vaccines support the further development of ETEC Vaccine 1 as an effective ETEC vaccine. Furthermore, the broad applicability of the *spi*-VEC strain should allow the development of a multivalent live attenuated enteropathogen vaccines based on this technology.

In conclusion, this pilot human study has shown that a 10^8^ or 10^9^ dose of live attenuated *S. typhi* expressing ETEC LT antigen is well tolerated, and highly immunogenic, inducing humoral immune responses to the inserted antigen in around two-thirds of subjects immunized orally after one or two doses.

## Figures and Tables

**Table 1:** Number of patients reporting gastrointestinal adverse events considered related to the vaccine by severity

Symptoms	Mild	Moderate	Severe
10^8^ Dose (*n* = 12)			
All gastrointestinal disorders	9 (75)	3 (25)	1 (8)
Abdominal pain/discomfort	7 (58)	2 (17)	1 (8)
Anorexia	1 (8)	0 (0)	0 (0)
Constipation	1 (8)	1 (8)	0 (0)
Culture stool positive	0 (0)	0 (0)	0 (0)
Diarrhea	0 (0)	0 (0)	0 (0)
Flatulence	4 (33)	0 (0)	0 (0)
Nausea	1 (8)	0 (0)	0 (0)
Vomiting	0 (0)	0 (0)	0 (0)

10^9^ Dose (*n* = 24)			
All gastrointestinal disorders	18 (75)	2 (8)	0 (0)
Abdominal pain/discomfort	12 (50)	1 (4)	0 (0)
Anorexia	1 (4)	0 (0)	0 (0)
Constipation	6 (25)	0 (0)	0 (0)
Culture stool positive	1 (4)	0 (0)	0 (0)
Diarrhea	1 (4)	1 (4)	0 (0)
Flatulence	14 (58)	1 (4)	0 (0)
Nausea	4 (16)	0 (0)	0 (0)
Vomiting	1 (4)	0 (0)	0 (0)

Total of subjects recording any event, subjects may record events of more than one severity grade. Figures in parentheses indicate percentage of group at that dose level (12 subjects in 10^8^ CFU, and 24 subjects in 10^9^ CFU group). In the 10^9^ CFU group, two subjects did not receive a second immunization.

**Table 2:**
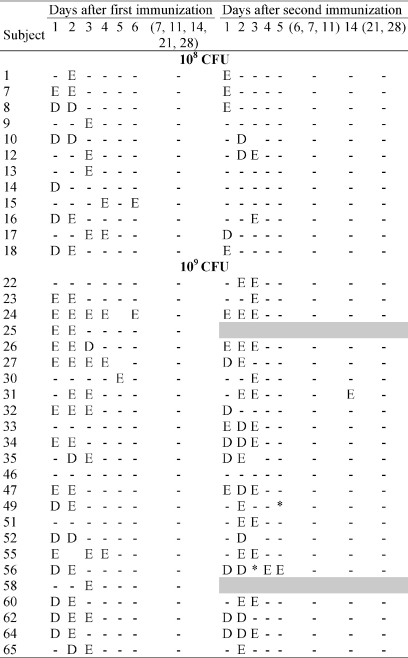
Fecal shedding of vaccine strain after immunization

D: vaccine growth on direct plating without requirement for enrichment; E: vaccine growth after enrichment only; –: no growth; *: sample not available. Grayed-out rows indicate subject did not receive second dose.

**Table 3:** Frequency of LT-B and LPS responses

	*n*	First immunization	*n*	Second immunization	First or Second Immunization
Number of positive responders to *S. typhi* LPS					
10^8^ CFU					
ELISA	1212	10 (83)	12	9 (75)	10 (83)
ASCs		11 (92)	12	4 (33)	11 (92)

10^9^ CFU					
ELISA	24	19 (79)	22	16 (73)	22 (92)
ASCs	24	21 (86)	22	8 (36)	22 (92)

Number of positive responders to LT-B					
10^8^ CFU					
ELISA	12	5 (42)	12	5 (42)	7 (58)
ASCs	12	0	12	1 (8)	1 (8)

10^9^ CFU					
ELISA	24	10 (42)	22	10 (45)	15 (63)
ASCs	24	3 (13)	22	0	3 (13)

Number of subjects with a positive response at any time point after each immunization as defined for each assay (percentage of group in parentheses—note in the 10^9^ group two subjects did not receive a second immunization). For ELISA positive response defined as ≥4-fold increase in serum IgG antibody titer between each time point and Day 0. For ELISPOT positive response defined as ≥10 ASCs/10^6^ PBMC secreting antigen-specific IgA at Day 7, 11 or 14 after each immunization.
